# Association of preoperative Pfirrmann grade with 12-month outcomes after interlaminar full-endoscopic lumbar discectomy with successful annular suture repair: a single-center retrospective cohort study

**DOI:** 10.3389/fsurg.2026.1924468

**Published:** 2026-10-08

**Authors:** Lei Zhou, Haoyun Huang, Song Zhu, Xin Chen, Yunchuan Gou, Jikun Sun, Pin Xie, Yi Zhou, Rigao Chen

**Affiliations:** Department of Orthopedics, Hospital of Chengdu University of Traditional Chinese Medicine, Chengdu, Sichuan, China

**Keywords:** annular suture repair, full-endoscopic lumbar discectomy, lumbar disc herniation, pfirrmann grading, propensity score, symptomatic recurrence

## Abstract

**Background:**

Whether advanced disc degeneration affects outcomes after annular suture repair remains uncertain. This study evaluated the association of Pfirrmann grade IV with 12-month outcomes and MRI-confirmed symptomatic recurrence among selected patients undergoing interlaminar full-endoscopic lumbar discectomy with successful annular suture repair.

**Methods:**

This single-center retrospective cohort included 226 patients treated between May 2023 and March 2025. Patients were classified as Pfirrmann grades I–III (*n* = 151) or grade IV (*n* = 75). The primary estimands were ATT-IPTW-adjusted between-group differences in VAS-back, VAS-leg, and ODI at 12 months. Weighted generalized estimating equations assessed longitudinal trajectories. Modified MacNab outcomes and MRI-confirmed symptomatic recurrence were secondary outcomes; postoperative MRI was symptom-triggered.

**Results:**

At 12 months, adjusted grade IV-minus-grades I–III differences were 0.070 for VAS-back (95% CI, −0.270–0.409), −0.005 for VAS-leg (95% CI, −0.334–0.325), and 0.297 for ODI (95% CI, −1.180–1.774). Results were consistent in propensity-score-matched, grades III–IV restricted, and ordinal sensitivity analyses. Excellent/good modified MacNab outcomes occurred in 93.4% and 92.0% of patients, respectively. MRI-confirmed symptomatic recurrence occurred in 6/151 and 2/75 patients. Recurrence analyses were exploratory because only eight events occurred.

**Conclusions:**

Among selected patients with successful annular suture repair, Pfirrmann grade IV was not associated with a clinically important disadvantage in 12-month pain or functional outcomes. No increased recurrence signal was observed, but the study cannot determine whether Pfirrmann grade affects surgical candidacy, repairability, or successful closure.

## Introduction

Lumbar disc herniation (LDH) is a common degenerative spinal disorder that may lead to low back pain, radicular pain, sensory disturbance, and functional impairment (1).

Full-endoscopic lumbar discectomy has become an important minimally invasive surgical option for single-level LDH because of its limited soft-tissue trauma, rapid recovery, and reduced disruption of normal anatomical structures (2, 3). Despite its favorable decompression efficacy, conventional discectomy alone does not restore annular integrity. A persistent annular defect may serve as a pathway for re-extrusion of residual nucleus pulposus and may contribute to postoperative recurrence and residual low back pain (4).

To reduce the risk of recurrent herniation, annulus fibrosus suture repair has gradually been introduced into clinical practice. This technique mechanically approximates the annular defect and is intended to restore barrier function, restrict re-extrusion of residual nucleus pulposus, and partially preserve the local biomechanical environment of the intervertebral disc. However, the clinical efficacy of annular repair may be influenced by host tissue quality and intrinsic repair potential. The Pfirrmann grading system is a commonly used MRI-based method for assessing lumbar disc degeneration and provides a relatively objective reflection of disc hydration and structural change. Theoretically, advanced degeneration is associated with poorer tissue quality and impaired mechanical integrity, which may weaken suture-holding capacity and repair durability and thus affect postoperative outcomes (5, 6).

Existing studies have primarily examined whether annular closure or repair reduces recurrent herniation compared with discectomy alone. A separate prognostic question is whether advanced preoperative disc degeneration remains associated with worse postoperative outcomes among patients in whom annular suture repair is judged technically feasible and is successfully completed. This question is clinically relevant because Pfirrmann grade IV may reflect reduced disc hydration, poorer annular tissue quality, and a less favorable biomechanical environment, which could theoretically compromise the durability of a completed repair. However, outcomes observed in such a selected cohort cannot determine whether degeneration severity influences the probability of intraoperative repairability or technical success.

Evidence directly addressing postoperative prognosis according to degeneration severity in patients uniformly treated with full-endoscopic annular suture repair remains limited. Therefore, the present study evaluated whether preoperative Pfirrmann grade IV was associated with 12-month patient-reported outcomes and MRI-confirmed symptomatic recurrence after interlaminar full-endoscopic lumbar discectomy with successful annular suture repair. This study was designed as a prognostic cohort study within a surgically selected and successfully repaired population. It was not designed to compare annular suture repair with discectomy alone, assess postoperative structural annular healing, or determine whether Pfirrmann grade influences intraoperative repairability or technical success.

## Materials and methods

### Study design and patient selection

This was a single-center retrospective observational cohort study including patients with single-level LDH who underwent interlaminar full-endoscopic lumbar discectomy with successful annular suture repair at the Department of Orthopedics, Hospital of Chengdu University of Traditional Chinese Medicine, between May 2023 and March 2025. Follow-up data were collected through March 2026, and the database was locked on April 1, 2026. This study evaluated prognosis according to degeneration severity within a surgically selected cohort in which annular suture repair was successfully completed.

The study was conducted in accordance with the Declaration of Helsinki and was approved in June 2025 by the Ethics Committee of the Affiliated Hospital of Chengdu University of Traditional Chinese Medicine (Approval No. 2025KL-126). The May 2023 to March 2025 study period refers to the period during which the clinical records were generated as part of routine patient care. Formal research-related data extraction and statistical analysis were undertaken after ethics approval. All clinical data were de-identified before analysis. Because of the retrospective design and the use of de-identified clinical data, the requirement for written informed consent was waived by the Ethics Committee. Propensity score-based methods were used to reduce potential confounding. The study was reported in accordance with the Strengthening the Reporting of Observational Studies in Epidemiology (STROBE) statement.

Patients were identified from surgical records using a stepwise screening process. A total of 645 patients were identified during the study period. In 25 patients, postoperative records did not document annular suture repair. Their preoperative Pfirrmann grade distribution was grade I in 0 patients, grade II in 0, grade III in 16, grade IV in 9, and grade V in 0. The available retrospective records did not permit the specific reasons for absent documented repair to be reliably distinguished. These patients were therefore described separately and were not used to evaluate the association between Pfirrmann grade and intraoperative repairability or technical success. Among the 620 patients with documented successful annular suture repair, 116 were excluded because they were younger than 18 years or older than 65 years, leaving 504 patients for eligibility assessment. A further 224 patients were excluded sequentially according to the eligibility criteria: multilevel disease (*n* = 132), previous or revision lumbar surgery (*n* = 37), spondylolisthesis of grade I or higher (*n* = 17), lumbar instability or severe spinal stenosis (*n* = 24), far-lateral or extraforaminal disc herniation (*n* = 11), and severe nuclear calcification or posterior vertebral rim separation (*n* = 3). No patients were excluded because of missing preoperative MRI, preoperative Pfirrmann grade V, concurrent spinal infection, tumor, or fracture, or cauda equina syndrome requiring emergency surgery (all *n* = 0). The remaining 280 patients met the clinical and imaging eligibility criteria. Of these, 54 did not complete the required 12-month follow-up and were excluded from the primary complete-case analysis. Consequently, 226 patients were included in the final analytic cohort (Figure 1).

**Figure 1:**
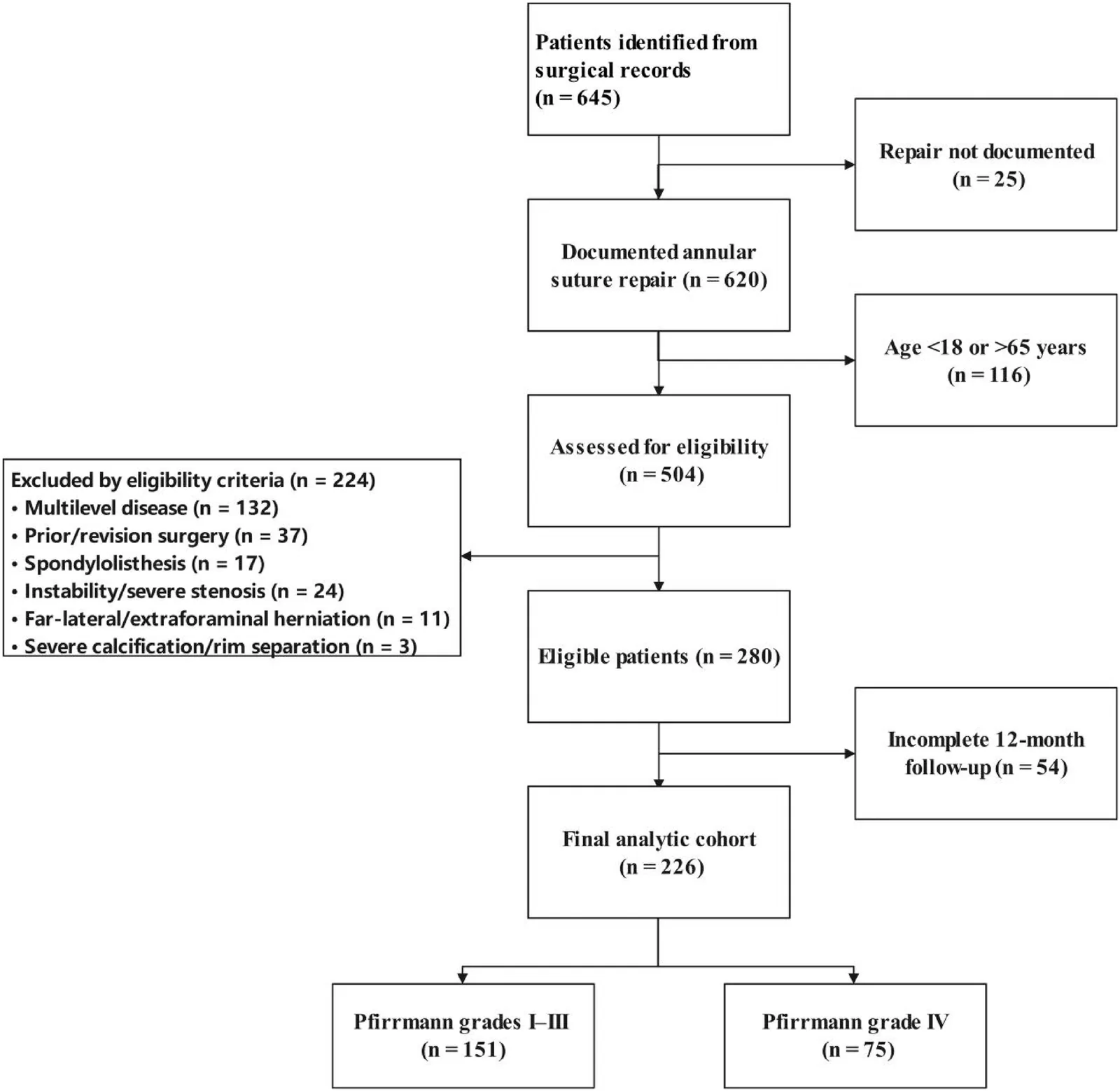
STROBE-style flow diagram of patient selection. Note. A total of 645 patients were identified from surgical records during the study period. Annular suture repair was not documented in 25 patients, and the specific intraoperative reasons could not be reliably retrieved. Among the 620 patients with documented successful annular suture repair, 116 were outside the predefined age range of 18–65 years. Of the remaining 504 patients, 224 were excluded according to the specified clinical and imaging eligibility criteria, leaving 280 eligible patients. No exclusions occurred because of missing preoperative MRI, Pfirrmann grade V degeneration, concurrent spinal infection, tumor, or fracture, or cauda equina syndrome requiring emergency surgery. Fifty-four patients did not complete the required 12-month follow-up, and 226 patients were included in the final complete-case analytic cohort, comprising 151 patients with Pfirrmann grades I–III and 75 patients with Pfirrmann grade IV.

### Inclusion and exclusion criteria

Inclusion criteria were as follows: (1) single-level LDH at L4/5 or L5/S1, with concordant clinical symptoms, neurological signs, and MRI or CT findings; (2) failure of conservative treatment for at least 6 weeks, except in patients with progressive neurological deficits or severe radicular pain constituting a clear surgical indication; (3) age between 18 and 65 years; (4) treatment with interlaminar full-endoscopic lumbar discectomy combined with documented successful annular suture repair; and (5) preoperative MRI demonstrating Pfirrmann grade I–IV degeneration at the index level. Complete clinical follow-up through 12 months was required for inclusion in the primary complete-case analysis. Some of these patients had partial postoperative VAS-back, VAS-leg, and ODI measurements before their last recorded follow-up; these measurements were retained for reporting data availability but were not included in the primary complete-case outcome analyses. MRI was obtained when symptomatic recurrence was clinically suspected; routine postoperative MRI was not part of the follow-up protocol.

Exclusion criteria were as follows: (1) multilevel lumbar disc disease; (2) previous or revision lumbar surgery; (3) lumbar spondylolisthesis of grade I or higher; (4) frank lumbar instability or severe spinal stenosis; (5) far-lateral or extraforaminal disc herniation; (6) severe nuclear calcification or posterior vertebral rim separation; (7) preoperative Pfirrmann grade V degeneration; (8) concurrent spinal infection, tumor, or fracture; and (9) cauda equina syndrome requiring emergency surgery.

### Imaging assessment and group allocation

All preoperative MRI examinations were independently reviewed by two spine surgeons, each with more than 10 years of experience in spine practice and lumbar MRI assessment. Both evaluators were blinded to the patients' clinical outcomes and to each other's initial ratings during the independent assessment. Preoperative lumbar MRI examinations were performed using conventional 1.5-T or 3.0-T scanners according to routine clinical imaging protocols. Pfirrmann grading was based on conventional sagittal T2-weighted images; synthetic MRI-derived images or quantitative synthetic MRI maps were not used. Disc degeneration at the index level was graded according to the original five-grade Pfirrmann classification using T2-weighted sagittal images. After completion of the independent assessments, discrepant ratings were resolved through joint re-evaluation and consensus. Only the final consensus grade was retained in the analytic dataset; the reader-specific initial ratings were not preserved in a form suitable for analysis. Consequently, formal inter-rater agreement statistics, including a weighted *κ* coefficient, could not be calculated. Additional preoperative imaging variables included Modic type, disc height index (DHI), and disc herniation morphology. Modic changes adjacent to the index disc were categorized as absent, type I, type II, or type III. DHI was measured on preoperative lumbar digital radiographs along the line connecting the centers of the vertebral bodies adjacent to the index disc and was calculated as the midline intervertebral disc height divided by the midline height of the cranial adjacent vertebral body. Disc herniation morphology was classified as protrusion, extrusion, or sequestration. The complete category-specific distributions were retained for descriptive reporting. Because Modic type III and sequestrated herniations were uncommon, Modic types II and III and, separately, extrusion and sequestration were combined only for propensity score estimation to avoid unstable estimates caused by sparse cells. The exact distribution of Pfirrmann grades was reported separately. Representative sagittal T2-weighted images of grade III and grade IV degeneration are shown in Figure 2. For the primary analysis, patients were classified as Pfirrmann grades I–III or grade IV. The primary exposure contrasted Pfirrmann grade IV with grades I–III. Because grades I and II included only 2 and 8 patients, respectively, grade III versus IV and ordinal grade I–IV analyses were performed as sensitivity analyses for pain and functional outcomes; recurrence analyses retained the binary exposure because only eight events occurred. Pfirrmann grade V discs were excluded because of severe disc-space collapse.

**Figure 2:**
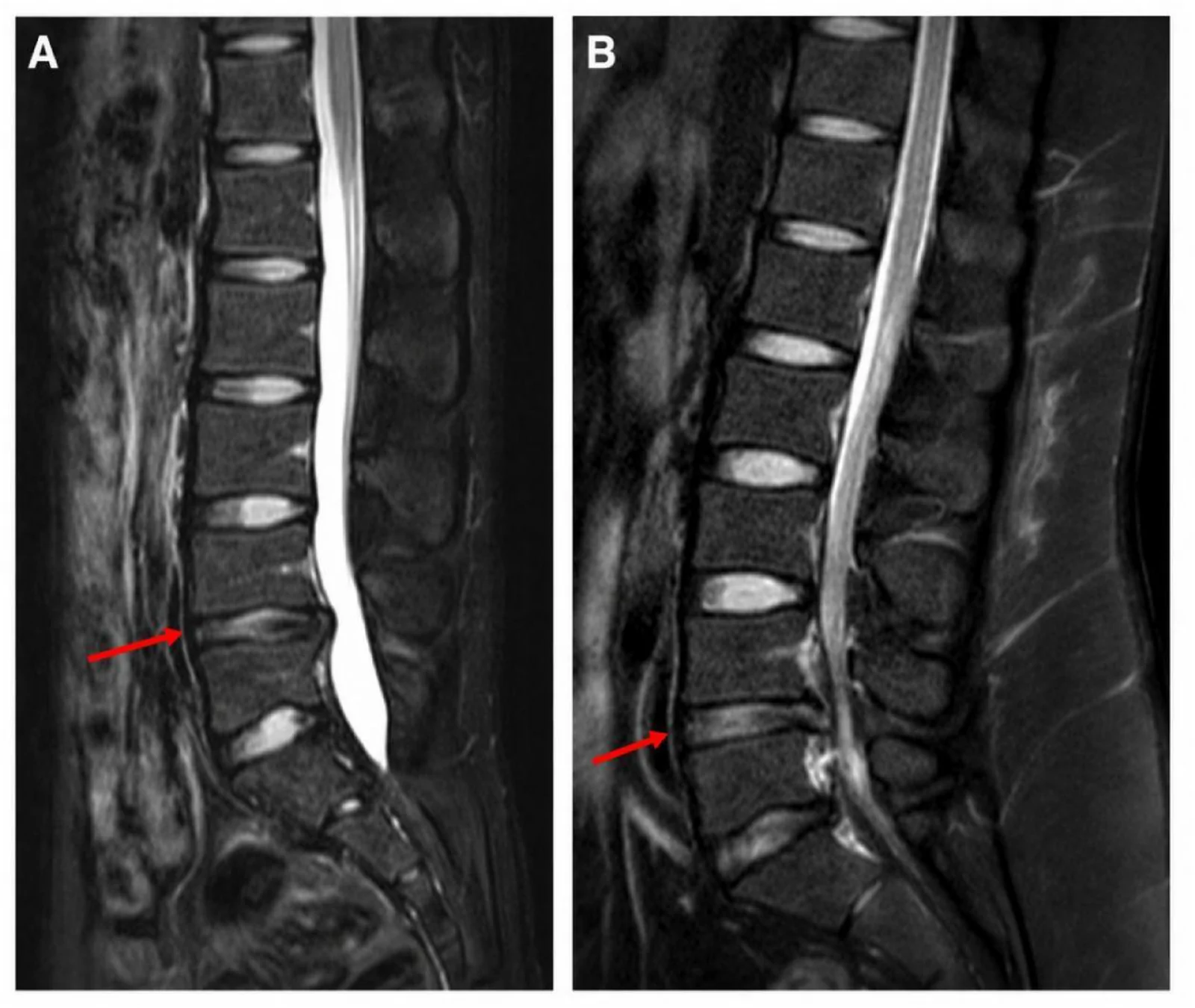
Representative preoperative sagittal T2-weighted MR images at the L4/5 index level. **(A)** A representative patient from the Pfirrmann grades I–III group, showing grade III degeneration. **(B)** A representative patient from the Pfirrmann grade IV group, showing grade IV degeneration.

### Surgical technique

All procedures were performed by a single senior spine surgeon using an interlaminar full-endoscopic approach under general anesthesia. With the patient in the prone position, a working channel was established under C-arm fluoroscopic guidance, and standardized decompression was performed under direct endoscopic visualization. For contained herniations, a limited annular incision was made at the site of maximal protrusion. For extruded or sequestered herniations, the existing defect was minimally enlarged only when necessary to remove loose nuclear fragments.

After decompression, repairability was assessed according to annular tissue quality and defect morphology, including whether the defect edges could be approximated without excessive tissue tearing and whether marked calcification or rigid rim separation was present. In repairable cases, a disposable annular suture device (Shanghai Minorth Medical Device Co., Ltd., China) was introduced through the working channel. All included patients underwent the same standardized single-needle, single-stitch linear suture procedure. Successful repair was defined as visible approximation of the defect without residual nerve-root compression or suture-related mechanical conflict.

Annular-defect size and precise location, Modic change extent, quantitative calcification severity, segmental mobility, amount of nucleus pulposus removed, and graded closure quality were not consistently retained in the operative records and could not be included as covariates. All patients received standardized perioperative management and postoperative rehabilitation.

### Outcome measures

VAS-back, VAS-leg, and ODI were recorded preoperatively and at 1, 3, 6, and 12 months postoperatively. The prespecified primary time point was 12 months, and the primary estimands were the ATT-IPTW-adjusted between-group differences in 12-month VAS-back, VAS-leg, and ODI scores, calculated as Pfirrmann grade IV minus grades I–III. Earlier assessments were used to describe longitudinal recovery. Operative time, length of hospital stay, the 12-month modified MacNab rating, symptomatic recurrence, and reoperation were secondary outcomes.

Follow-up assessments were conducted by trained research personnel who were blinded to preoperative Pfirrmann grade. Outpatient and telephone assessments used the same structured form, item wording, scale anchors, scoring instructions, and data-entry rules. During telephone follow-up, VAS, ODI, and modified MacNab items were administered verbally using the same definitions as during outpatient assessment.

Symptomatic recurrence was defined as recurrent ipsilateral radicular pain consistent with the preoperative symptoms after a symptom-free interval of at least 1 month, together with MRI confirmation of recurrent disc herniation at the index level. Contralateral same-level herniation was classified as a separate new event rather than primary ipsilateral recurrence. Routine postoperative MRI was not performed; MRI was obtained only when recurrent symptoms suggested possible recurrence. Accordingly, the endpoint represented MRI-confirmed symptomatic recurrence rather than asymptomatic radiographic recurrence. Individual recurrence times and recurrence-related reoperations were recorded.

MRI examinations obtained for suspected recurrence were independently evaluated by the same two experienced spine surgeons who performed the preoperative grading. Disagreements were resolved by consensus. The retrospective database did not contain standardized variables for dural tear, neurological deterioration, paresthesia or dysesthesia, infection, hematoma, or suture-related nerve-root compression. Reliable complication-specific incidence rates and between-group safety comparisons therefore could not be reconstructed.

### Statistical analysis

Analyses were performed using SPSS version 26.0 and R. Continuous variables were summarized as mean ± standard deviation or median (interquartile range) and compared using the independent-samples t test or Mann–Whitney U test, as appropriate. Categorical variables were summarized as counts and percentages and compared using the chi-square, Fisher exact, or Fisher–Freeman–Halton exact test.

The propensity score was estimated using multivariable logistic regression, with Pfirrmann grade IV as the exposure. Covariates included age, sex, body mass index, symptom duration, smoking, diabetes, hypertension, surgical level, herniation laterality, disc height index, herniation morphology, Modic type, and preoperative VAS-back, VAS-leg, and ODI. Continuous variables were entered as linear terms and categorical variables as indicator variables. Because of sparse categories, herniation morphology was modeled as protrusion versus extrusion/sequestration and Modic status as absent, type I, or type II/III.

For ATT estimation, patients with grade IV received a weight of 1, whereas patients with grades I–III received a weight of eᵢ/(1−eᵢ), where eᵢ was the estimated probability of grade IV. Raw weights were used without stabilization, normalization, trimming, or truncation. Overlap and positivity were assessed using group-specific propensity-score distributions, the empirical common-support interval, and weight distributions. Balance was assessed using absolute standardized mean differences, with values below 0.10 considered adequate. Effective sample size was calculated as (*Σ*wᵢ)^2^/*Σ*wᵢ^2^. Overlap and weight plots, a Love plot, and covariate-specific balance results are presented in Supplementary Figure S1 and Supplementary Table S1.

As a sensitivity analysis, 1:1 optimal propensity-score matching without replacement was performed using the same propensity model. Matching minimized the absolute distance on the logit propensity score, applied a caliper of 0.20 standard deviations of the logit propensity score (0.213 on the logit scale), and required exact matching on the collapsed herniation morphology category. Unmatched patients were excluded only from the matched analysis. Matched outcomes were estimated from within-pair differences, with confidence intervals and *P* values calculated using paired t inference.

The primary estimands were ATT-IPTW-adjusted differences in VAS-back, VAS-leg, and ODI at 12 months. Repeated measurements at baseline and 1, 3, 6, and 12 months were analyzed using ATT-weighted Gaussian generalized estimating equations with patient-level clustering, categorical time, and an exchangeable working correlation structure. Models included group, time, and group-by-time interaction terms. Patient-clustered sandwich standard errors accounted for repeated measurements and heteroskedasticity under weighting, with estimated ATT weights treated as fixed. The empirical covariance matrix was multiplied by (N/(N−q)) to reduce finite-sample bias, where (*N* = 226) patient clusters and (q = 10) regression coefficients. Weighted marginal means and grade IV-minus-grades I–III contrasts were estimated at 12 months. Group-by-time interactions were supportive trajectory analyses and were not used as the primary test of the 12-month endpoint.

ATT-weighted linear regression of 12-month improvement scores was performed as a sensitivity analysis. Because these models contained one observation per patient, HC3 heteroskedasticity-consistent standard errors were used, with standard-normal Wald confidence intervals and two-sided *P* values. Additional analyses restricted the cohort to grades III and IV, with re-estimation of the propensity score, and modeled Pfirrmann grade as an ordinal exposure in covariate-adjusted linear regression. The same HC3 covariance estimator and standard-normal Wald inference were used for the restricted ATT-weighted and adjusted ordinal models. Separate estimates for grades I and II were not calculated because these groups contained only 2 and 8 patients, respectively.

Recurrence analyses were exploratory because only eight events occurred. The ATT-weighted 12-month risk difference was estimated using a weighted linear probability model with heteroskedasticity-robust sandwich standard errors. For Kaplan–Meier analysis, surgery was time zero, the event was the first MRI-confirmed ipsilateral symptomatic recurrence, and event-free patients were administratively censored at 12 months. Curves were compared using the log-rank test.

There were no missing covariates or outcomes within the 226-patient complete-case cohort. Available baseline characteristics were compared between included patients and the 54 otherwise eligible patients who did not complete 12-month follow-up; preoperative VAS-back, VAS-leg, and ODI were available for all 54 non-completing patients. Available and missing longitudinal observations were reported at each assessment point, and no missing outcomes were imputed. All tests were two-sided, with *P* < 0.05 considered statistically significant. No formal sample-size calculation or non-inferiority margin was prespecified.

## Results

### Patient selection and comparison with patients who did not complete 12-month follow-up

Among 25 patients without documented annular suture repair, 16 had Pfirrmann grade III and 9 had grade IV degeneration; none had grade I, II, or V degeneration. Because the operative records did not reliably distinguish repair not attempted, technical unsuitability, or unsuccessful attempted repair, these patients were reported separately and were not included in analyses of repairability or technical success.

Among 280 patients who met the clinical and imaging eligibility criteria, 54 (19.3%) did not complete 12-month follow-up, leaving 226 patients in the complete-case cohort. Included and non-completing patients had similar available characteristics, including age, sex, L4/5 surgery, and grade IV prevalence (absolute SMD for grade IV = 0.077; Supplementary Table S2). Their Pfirrmann grade I/II/III/IV distributions were 2/8/141/75 and 0/3/35/16, respectively. Preoperative VAS-back (5.04 ± 0.93 vs. 5.04 ± 0.64; *P* = 0.988), VAS-leg (7.08 ± 1.13 vs. 7.06 ± 1.04; *P* = 0.880), and ODI (51.59 ± 8.72 vs. 50.22 ± 7.46; *P* = 0.244) were available for all 226 included patients and all 54 non-completing patients and did not differ significantly between cohorts. Other baseline characteristics were not consistently available; therefore, selection bias associated with complete-case restriction cannot be excluded.

### Baseline characteristics and covariate balance

The cohort included 2 patients with Pfirrmann grade I (0.9%), 8 with grade II (3.5%), 141 with grade III (62.4%), and 75 with grade IV (33.2%); 151 patients therefore comprised the grades I–III group. Before weighting, grade IV patients were older, more frequently female, and more likely to have Modic changes (all *P* ≤ 0.011). Disc height index and herniation morphology did not differ significantly between groups. Complete baseline distributions are presented in Table 1. The largest unweighted imbalances involved age (SMD = 0.623), Modic changes (SMD = 0.415), and sex (SMD = 0.370).

**Table 1:** Baseline demographic, clinical, and imaging characteristics before weighting.

Characteristic	Pfirrmann I–III (*n* = 151)	Pfirrmann IV (*n* = 75)	SMD	*P* value
Age, years	47.0 (36.0–54.0)	54.0 (47.5–59.5)	0.623	<0.001
Female sex, *n* (%)	57 (37.7)	42 (56.0)	0.370	0.011
BMI, kg/m^2^	24.76 ± 3.34	24.48 ± 3.48	0.084	0.554
Current smoker, *n* (%)	24 (15.9)	12 (16.0)	0.003	1.000
Diabetes mellitus, *n* (%)	7 (4.6)	6 (8.0)	0.138	0.366
Hypertension, *n* (%)	17 (11.3)	14 (18.7)	0.208	0.151
Duration of current symptomatic episode, months	3.00 (1.00–11.00)	3.00 (0.69–12.00)	0.043	0.655
Index level L4/5, *n* (%)	91 (60.3)	43 (57.3)	0.059	0.774
Right-sided herniation, *n* (%)	63 (41.7)	30 (40.0)	0.035	0.886
Disc height index	0.356 ± 0.079	0.350 ± 0.085	0.073	0.609
Any Modic change, *n* (%)	32 (21.2)	30 (40.0)	0.415	0.004
Modic type, *n* (%)				0.016
Absent	119 (78.8)	45 (60.0)	0.415	
Type I	5 (3.3)	5 (6.7)	0.154	
Type II	26 (17.2)	24 (32.0)	0.346	
Type III	1 (0.7)	1 (1.3)	0.067	
Herniation morphology, *n* (%)				0.410
Protrusion	98 (64.9)	54 (72.0)	0.152	
Extrusion	50 (33.1)	21 (28.0)	0.111	
Sequestration	3 (2.0)	0 (0.0)	0.201	
Preoperative VAS-back	5.09 ± 0.90	4.92 ± 0.97	0.184	0.199
Preoperative VAS-leg	7.05 ± 1.20	7.13 ± 0.98	0.073	0.591
Preoperative ODI	51.74 ± 8.99	51.31 ± 8.20	0.050	0.721

Continuous variables are presented as mean ± SD or median (interquartile range), as appropriate; categorical variables are presented as *n* (%). *P* values are unadjusted and were calculated using the Mann–Whitney U test for age and symptom duration, Welch's *t*-test for other continuous variables, Fisher's exact test for binary variables, and the Fisher–Freeman–Halton exact test for multicategory variables. SMDs are absolute standardized mean differences; category-specific SMDs for multicategory variables treat each displayed category as a binary indicator. There were no missing values in the 226-patient analytic cohort. BMI, body mass index; DHI, disc height index; ODI, Oswestry Disability Index; SD, standard deviation; SMD, standardized mean difference; VAS, visual analogue scale.

After expanded ATT-IPTW, all modeled covariates achieved an absolute SMD below 0.10, including disc height index, herniation morphology, and each modeled Modic category. The largest residual imbalance was for diabetes (SMD = 0.070). Covariate-specific results are presented in Supplementary Table S1. This balance applies only to measured variables and does not eliminate residual confounding from unrecorded anatomical or intraoperative factors.

### Propensity score and weight diagnostics

The estimated propensity scores ranged from 0.018 to 0.729 in the Pfirrmann grades I–III group and from 0.049 to 0.862 in the grade IV group. The empirical common-support interval was 0.049–0.729 and included 143 of 151 patients (94.7%) in the grades I–III group and 70 of 75 patients (93.3%) in the grade IV group, indicating substantial overlap, although limited non-overlap remained in the distribution tails.

All patients in the grade IV group received an ATT weight of 1.000. Among patients with Pfirrmann grades I–III, the weights ranged from 0.019 to 2.693, with a median of 0.313 and an interquartile range of 0.151–0.676. The sum of the weights was approximately 75.1 in the grades I–III group and 75.0 in the grade IV group. Because no markedly extreme weights were observed, no weight truncation or trimming was applied. The weighted effective sample size was 151.2 overall, comprising 76.2 effective patients in the grades I–III group and 75.0 in the grade IV group. Propensity-score overlap, ATT weight distributions, and a Love plot of covariate balance before and after weighting are presented in Supplementary Figure S1.

### Longitudinal clinical outcomes

All 226 patients in the complete-case cohort had VAS-back, VAS-leg, and ODI data at baseline and at 1, 3, 6, and 12 months. Among the 280 patients meeting the clinical and imaging criteria before complete-case restriction, complete data for all three outcomes were available for 280, 252, 243, 235, and 226 patients at baseline and at 1, 3, 6, and 12 months, respectively; the corresponding numbers missing were 0, 28, 37, 45, and 54.

Both groups showed substantial postoperative improvements in pain and function (overall time effects, all *P* < 0.001; Table 2). The supportive group-by-time interactions were not statistically significant for VAS-back (*P* = 0.357), VAS-leg (*P* = 0.464), or ODI (*P* = 0.962). These interactions describe overall recovery trajectories and do not replace the prespecified 12-month contrasts reported below.

**Table 2:** Longitudinal clinical outcomes and ATT-IPTW-weighted estimates at the 12-month primary endpoint.

Panel A. Unweighted descriptive longitudinal outcomes and supportive weighted GEE tests.						
Outcome	Time point	Pfirrmann I⁠–⁠III	Pfirrmann IV	Unadjusted P	ATT-IPTW GEE P (time)	ATT-IPTW GEE P (group × time)
VAS-back	Baseline	5.09 ± 0.90	4.92 ± 0.97	0.199	<0.001	0.357
	1 month	2.56 ± 2.06	3.17 ± 2.27	0.052		
	3 months	1.89 ± 1.69	2.03 ± 1.76	0.589		
	6 months	1.13 ± 0.87	1.16 ± 0.92	0.788		
	12 months	2.07 ± 1.16	1.99 ± 1.05	0.605		
VAS-leg	Baseline	7.05 ± 1.20	7.13 ± 0.98	0.591	<0.001	0.464
	1 month	2.91 ± 2.19	3.44 ± 2.37	0.110		
	3 months	1.99 ± 1.86	1.92 ± 1.49	0.771		
	6 months	1.28 ± 1.13	1.44 ± 0.96	0.263		
	12 months	1.28 ± 1.24	1.21 ± 1.13	0.695		
ODI	Baseline	51.74 ± 8.99	51.31 ± 8.20	0.721	<0.001	0.962
	1 month	22.38 ± 11.46	24.99 ± 10.07	0.083		
	3 months	20.90 ± 7.71	22.08 ± 6.60	0.234		
	6 months	13.32 ± 5.04	13.52 ± 4.84	0.778		
	12 months	11.55 ± 6.28	11.57 ± 4.55	0.974		

Panel B. ATT-IPTW-weighted marginal estimates at 12 months				
Outcome	Pfirrmann I–III weighted marginal mean	Pfirrmann IV weighted marginal mean	Adjusted difference, IV minus I–III (95% CI)	*P* value
VAS-back	1.917	1.987	0.070 (−0.270 to 0.409)	0.687
VAS-leg	1.218	1.213	−0.005 (−0.334 to 0.325)	0.977
ODI	11.276	11.573	0.297 (−1.180 to 1.774)	0.692

All 226 patients in the final complete-case analytic cohort contributed VAS-back, VAS-leg, and ODI measurements at baseline and at 1, 3, 6, and 12 months. Among the 280 patients who met the clinical and imaging eligibility criteria before complete-case restriction, the corresponding numbers with complete VAS-back, VAS-leg, and ODI data were 280, 252, 243, 235, and 226 at baseline, 1, 3, 6, and 12 months, respectively. Panel A presents unweighted descriptive values as mean ± SD. Longitudinal model-based *P* values were obtained from ATT-IPTW-weighted generalized estimating equation models with a Gaussian distribution, an exchangeable working correlation structure, and robust standard errors. Time was modeled as a categorical variable. Reported confidence intervals and Wald *P* values incorporated the N/(N−q) finite-sample correction to the empirical sandwich covariance matrix, with *N* = 226 patient clusters and q = 10 fixed-effect coefficients. Panel B presents model-based weighted marginal means and the prespecified contrasts at the 12-month primary time point. Adjusted differences were calculated as Pfirrmann grade IV minus Pfirrmann grades I–III. ATT-IPTW, inverse probability of treatment weighting for the average treatment effect in the treated; CI, confidence interval; GEE, generalized estimating equation; ODI, Oswestry Disability Index; SD, standard deviation; VAS, visual analogue scale.

### Twelve-month endpoint outcomes

At 12 months, ATT-weighted marginal means for VAS-back were 1.917 in the grades I–III group and 1.987 in the grade IV group, giving an adjusted grade IV-minus-grades I–III difference of 0.070 points (95% CI, −0.270–0.409; *P* = 0.687). Corresponding VAS-leg means were 1.218 and 1.213, with a difference of −0.005 points (95% CI, −0.334–0.325; *P* = 0.977). ODI means were 11.276 and 11.573, with a difference of 0.297 points (95% CI, −1.180–1.774; *P* = 0.692).

The confidence intervals were substantially narrower than published minimum clinically important difference benchmarks for VAS-back, VAS-leg, and ODI, although these benchmarks were not prespecified non-inferiority margins (7). Operative time and hospital stay were similar between groups, and excellent/good modified MacNab outcomes occurred in 93.4% and 92.0%, respectively (*P* = 0.784; Table 3). Because complications could not be ascertained consistently, no comparative safety analysis was performed.

**Table 3:** Perioperative and 12-month secondary outcomes.

Outcome	Pfirrmann I–III (*n* = 151)	Pfirrmann IV (*n* = 75)	*P* value
Operative time, min	69.00 (55.00–83.00)	70.00 (55.00–80.00)	0.961
Length of stay, days	4.00 (3.50–5.00)	4.00 (4.00–5.50)	0.166
12-month modified MacNab: Excellent, *n* (%)	117 (77.5)	59 (78.7)	0.510
Good, *n* (%)	24 (15.9)	10 (13.3)	
Fair, *n* (%)	7 (4.6)	6 (8.0)	
Poor, *n* (%)	3 (2.0)	0 (0.0)	
Excellent/Good outcome, *n* (%)	141 (93.4)	69 (92.0)	0.784
Symptomatic recurrence within 12 months, *n* (%)	6 (4.0)	2 (2.7)	1.000
Individual time to recurrence, months^a^	2, 3, 3, 9, 12, 12	4, 6	
Reoperation for recurrence, *n* (%)	2 (1.3)	0 (0.0)	1.000

Modified MacNab outcomes and symptomatic recurrence status through 12 months were available for all 226 patients. Continuous variables are presented as median (interquartile range); categorical variables are presented as *n* (%). The *P* value for modified MacNab grading refers to the overall four-category distribution. ^a^Individual event times are reported because only eight MRI-confirmed symptomatic recurrences occurred. Symptomatic recurrence was defined as recurrent ipsilateral radicular pain at the index level after at least 1 month of symptomatic relief, confirmed by magnetic resonance imaging.

### Symptomatic recurrence

Eight MRI-confirmed symptomatic recurrences occurred: 6/151 (4.0%) in the grades I–III group and 2/75 (2.7%) in the grade IV group. Individual recurrence times were 2, 3, 3, 9, 12, and 12 months in the grades I–III group and 4 and 6 months in the grade IV group. Kaplan–Meier curves were largely overlapping (log-rank *P* = 0.619; Figure 3).

**Figure 3:**
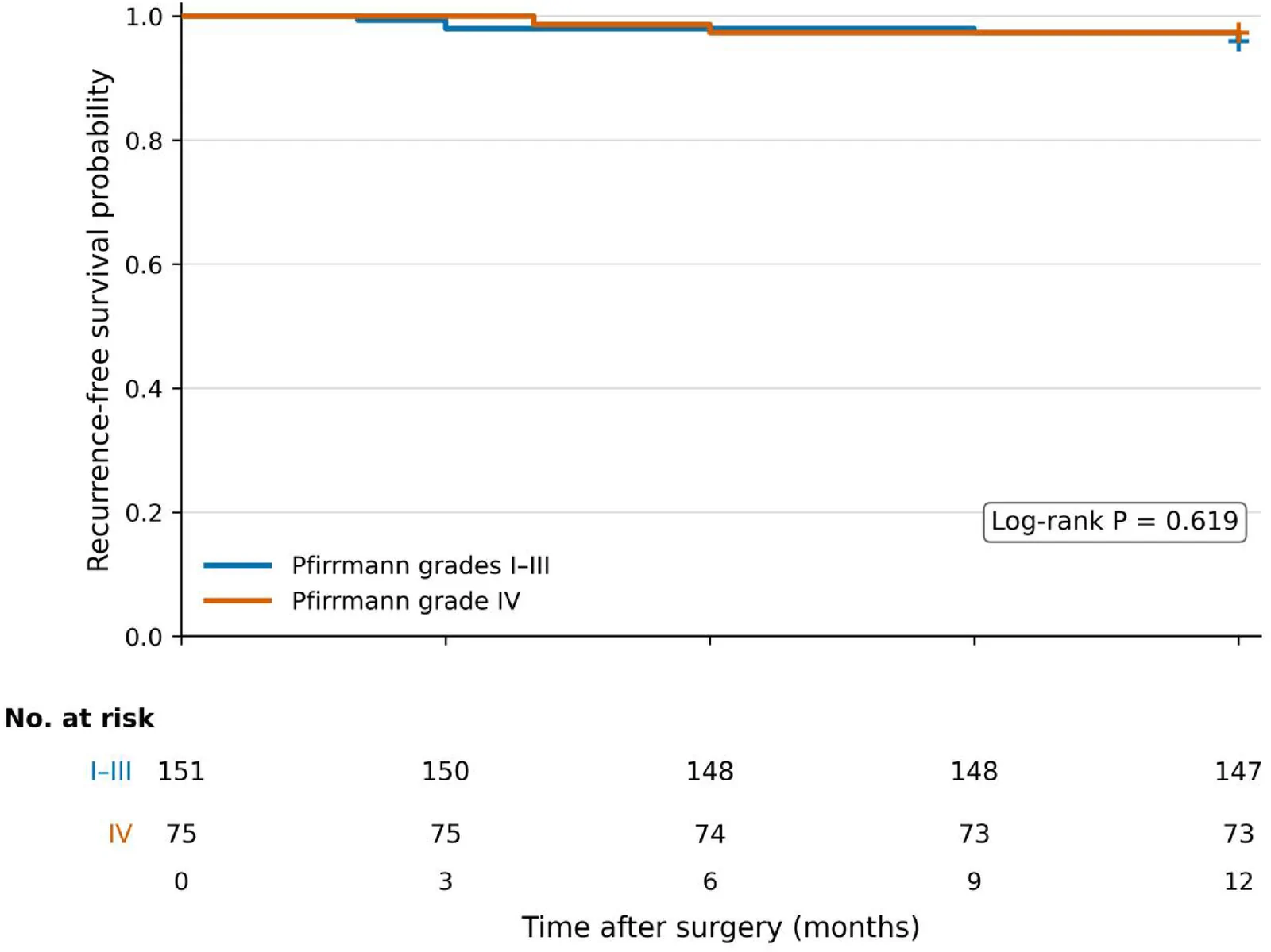
Kaplan–meier curves of recurrence-free survival stratified by pfirrmann grade. Note. There was no significant difference in recurrence-free survival between the Pfirrmann grades I–III group and the Pfirrmann grade IV group (log-rank *P* = 0.619).

ATT-weighted 12-month risks were 5.7% and 2.7%, respectively, corresponding to a grade IV-minus-grades I–III risk difference of −3.0 percentage points (95% CI, −9.1–3.0 percentage points; *P* = 0.323). Two patients underwent reoperation, both in the grades I–III group. Thus, no increased recurrence signal was observed in the grade IV group; however, with only eight events and symptom-triggered MRI, these analyses remain exploratory and cannot exclude a clinically relevant increase or decrease in risk.

### Sensitivity and robustness analyses

ATT-weighted improvement-score analyses showed no association between grade IV and improvement in VAS-back, VAS-leg, or ODI (Table 4). Results were materially unchanged after restriction to grades III and IV and after modeling Pfirrmann grade as an ordinal exposure. The small numbers of grade I and II patients precluded reliable grade-specific estimates.

**Table 4:** Sensitivity analyses based on 12-month improvement scores.

Analysis	Outcome	*β*	95% CI	*P* value
Primary ATT-IPTW: Pfirrmann IV vs. I–III	*Δ*VAS-back	−0.015	−0.479 to 0.448	0.948
	*Δ*VAS-leg	−0.017	−0.457 to 0.422	0.938
	*Δ*ODI	−0.005	−2.900 to 2.889	0.997
Restricted ATT-IPTW: Pfirrmann IV vs. III	*Δ*VAS-back	−0.014	−0.473 to 0.446	0.953
	*Δ*VAS-leg	−0.018	−0.459 to 0.423	0.936
	*Δ*ODI	−0.042	−2.937 to 2.852	0.977
Adjusted ordinal model: per one-grade increase	*Δ*VAS-back	−0.013	−0.317 to 0.291	0.931
	*Δ*VAS-leg	0.017	−0.277 to 0.310	0.912
	*Δ*ODI	0.010	−1.367 to 1.386	0.989
PSM: Pfirrmann IV vs. I–III	*Δ*VAS-back	0.088	−0.371 to 0.548	0.703
	*Δ*VAS-leg	0.250	−0.335 to 0.835	0.396
	*Δ*ODI	0.735	−3.010 to 4.480	0.696

Improvement scores were calculated as the preoperative value minus the 12-month postoperative value; therefore, a positive value indicates greater improvement. For the primary and restricted ATT-IPTW analyses, β represents the weighted difference in improvement for Pfirrmann grade IV relative to the indicated reference group. For the ordinal analysis, β represents the adjusted difference associated with each one-grade increase in Pfirrmann degeneration. For the propensity score-matched analysis, β represents the mean within-pair difference in improvement between Pfirrmann grade IV and grades I–III; 95% confidence intervals and *P* values were calculated using paired inference. The propensity score models incorporated demographic, clinical, and imaging covariates, including DHI, herniation morphology, Modic type, and preoperative outcome scores. ATT-IPTW, inverse probability of treatment weighting for the average treatment effect in the treated; CI, confidence interval; DHI, disc height index; ODI, Oswestry Disability Index; PSM, propensity score matching; VAS, visual analogue scale.

Propensity-score matching yielded 68 matched pairs; 7 grade IV and 83 grades I–III patients remained unmatched. All modeled covariates achieved absolute SMDs below 0.10 after matching. In the matched cohort, grade IV-minus-grades I–III differences at 12 months were −0.059 for VAS-back (95% CI, −0.388–0.270; *P* = 0.722), −0.147 for VAS-leg (95% CI, −0.529–0.235; *P* = 0.444), and −0.118 for ODI (95% CI, −2.043–1.808; *P* = 0.903). These estimates were consistent with the primary ATT-IPTW results.

## Discussion

This study examined whether preoperative Pfirrmann grade IV was associated with prognosis among selected patients who underwent successful interlaminar full-endoscopic lumbar discectomy with annular suture repair. Within this cohort, grade IV was not associated with clearly worse 12-month pain, functional, or modified MacNab outcomes than grades I–III. No increased signal of MRI-confirmed symptomatic recurrence was observed, but this finding remains exploratory because only eight events occurred.

At the prespecified 12-month endpoint, adjusted differences in VAS-back, VAS-leg, and ODI were close to zero, and their confidence intervals remained substantially within published minimum clinically important difference estimates of 1.2, 1.6, and 12.8 points, respectively (7). Because these thresholds were developed mainly for interpreting within-patient improvement and were not prespecified as formal non-inferiority margins, they provide clinical context rather than proof of equivalence. The findings therefore provide no evidence of a clinically important 12-month disadvantage associated with grade IV within this selected cohort. One possible explanation is that, after technically successful decompression and annular closure, 12-month pain and functional recovery may be influenced more strongly by adequate neural decompression and control of the annular defect than by preoperative disc signal characteristics alone. Moreover, Pfirrmann grade reflects overall disc degeneration but does not directly measure annular-edge quality, defect geometry, suture-holding strength, or closure integrity. The small between-group differences should therefore be interpreted as indicating limited prognostic discrimination of grade IV within this selected cohort, rather than an absence of biological differences between degeneration grades.

These findings do not demonstrate that annular suture repair reverses degeneration, restores annular integrity on postoperative imaging, or prevents asymptomatic radiographic recurrence. They apply only to selected patients in whom repair was successfully completed and cannot determine whether Pfirrmann grade IV influences surgical candidacy, intraoperative repairability, or technical success.

The primary grades I–III versus grade IV classification was used because only 2 and 8 patients had grades I and II, respectively, precluding stable grade-specific estimates. Results remained materially unchanged when the cohort was restricted to grades III and IV, when Pfirrmann grade was modeled ordinally, and when 68 propensity-score-matched pairs were analyzed. These findings reduce concern that the primary result was caused by pooling grades I–III, although the small grade I and II subgroups still prevent reliable estimation of individual or nonlinear grade-specific associations.

Previous studies suggest that the principal benefit of annular closure may be reduced symptomatic reherniation and reoperation rather than persistent additional improvement in pain or function. Thomé et al. reported that a bone-anchored annular closure device reduced symptomatic reherniation and reoperation at 5 years among patients with large annular defects (8). However, the strongest evidence concerns bone-anchored devices, whereas direct evidence for linear suture repair remains limited; these results therefore cannot be directly extrapolated to the technique used in the present study (9).

Endoscopic suture-repair studies have generally reported technical feasibility and favorable short-term outcomes. Peng et al. reported a modified MacNab excellent/good rate of 93.55% and a lower recurrence rate than full-endoscopic discectomy alone (10). Li et al. reported satisfactory 1-year outcomes after full-endoscopic annular suture repair (5), and Wang et al. found that the technique was feasible and might reduce recurrence without a consistent additional advantage in pain or functional recovery (11). The present excellent/good rate of more than 92% and overall symptomatic recurrence rate of 3.5% were broadly within the range of previous reports (10–12). However, differences in study design, selection criteria, comparator groups, and recurrence definitions preclude claims of superiority. Importantly, unlike previous studies comparing annular repair with discectomy alone, the present study compared degeneration grades within a uniformly treated and successfully repaired cohort. It therefore does not estimate the treatment effect of annular repair itself; rather, it addresses whether grade IV retains prognostic value after patient selection and successful closure.

Both groups improved rapidly after surgery, and the small numerical differences during early follow-up diminished thereafter. VAS-back increased mildly at 12 months in both groups but remained substantially below baseline; similar minor fluctuations during postoperative recovery have been described after lumbar discectomy (13). Because the increase occurred in both groups, it does not suggest a grade-specific effect, although longer follow-up is required to determine its clinical relevance (13).

The rationale for annular repair is to approximate the defect, restrict re-extrusion of residual nucleus pulposus, and partially preserve annular barrier function (14). Previous studies have suggested that greater disc degeneration may be associated with recurrence after endoscopic lumbar discectomy (15). Pfirrmann grade IV is itself heterogeneous and does not necessarily imply the severe disc-space collapse characteristic of grade V (6). In the present cohort, disc height index was similar between groups, grade V discs and overt instability were excluded, and all analyzed defects were successfully closed. These features may have narrowed the prognostic contrast between groups. This selection-based explanation should not be interpreted as meaning that all grade IV defects are technically repairable. Against this background, the absence of an increased recurrence signal in the grade IV group is relevant but should not be interpreted as showing that suture repair eliminates the biological or biomechanical effects of degeneration. Because the present cohort included only successfully repaired patients and the specific reasons for absent repair in 25 patients were unavailable, the study cannot assess whether degeneration affects repairability or technical success. Moreover, grade IV should not be equated with end-stage degeneration, and the findings should not be extrapolated to grade V discs, marked disc-space collapse, frank instability, severe calcification, or defects that cannot be effectively closed.

The grade IV group was older, included more women, and had a higher prevalence of Modic changes. These variables, together with disc height index and herniation morphology, were incorporated into the expanded propensity model and achieved adequate balance after ATT-IPTW. However, balance in measured covariates cannot eliminate residual confounding from unrecorded anatomical and intraoperative characteristics. Previous studies have suggested that Modic changes may be associated with poorer back-pain relief, functional recovery, or satisfaction after lumbar discectomy (16, 17). The absence of a clear adjusted disadvantage in the present cohort therefore should not be interpreted as showing that Modic changes are prognostically irrelevant; it may partly reflect patient selection, successful repair requirements, and control of measured confounders.

### Clinical implications

Among selected patients with successful annular suture repair, Pfirrmann grade IV alone did not identify a subgroup with clearly worse 12-month pain or functional outcomes. It therefore should not be used in isolation to predict prognosis after successful repair. This result does not establish that grade IV is irrelevant to surgical selection or repairability. Decisions should continue to integrate disc height, Modic and endplate changes, segmental stability, calcification, herniation morphology, annular tissue quality, and the ability to achieve effective defect closure.

### Limitations

This study has several limitations. First, its single-center retrospective design is susceptible to selection bias. Of 280 patients meeting the clinical and imaging criteria, 54 (19.3%) did not complete 12-month follow-up and were excluded from the primary complete-case analysis. Some had partial postoperative measurements, but these observations were not incorporated into the primary models, and no imputation or inverse-probability-of-censoring weighting was performed. Although age, sex, surgical level, Pfirrmann grade, and preoperative VAS-back, VAS-leg, and ODI were available for all 54 non-completing patients and were broadly similar to those in the included cohort, other baseline characteristics could not be compared consistently, and postoperative outcomes after loss to follow-up remained unobserved. Selection bias and reduced statistical efficiency therefore remain possible.

Second, follow-up was limited to 12 months, restricting assessment of late recurrence, reoperation, progressive degeneration, and persistent low back pain. Postoperative MRI was symptom-triggered rather than routinely obtained; consequently, asymptomatic radiographic recurrence, annular healing, disc-height change, and Modic or degenerative progression could not be evaluated.

Third, only eight MRI-confirmed symptomatic recurrences occurred. Recurrence estimates were therefore imprecise and exploratory. The primary definition required recurrent ipsilateral symptoms. Contralateral same-level herniation was considered a separate event, but the database did not contain a standardized variable for such events, so under-ascertainment or misclassification cannot be excluded.

Fourth, the primary dichotomization of grades I–III versus IV may have obscured nonlinear or grade-specific associations. Restricted and ordinal analyses were consistent with the primary findings, but the very small grade I and II groups prevented reliable individual estimates. Residual confounding also remains possible. Although disc height index, herniation morphology, and Modic type were included in the propensity model, annular-defect size and location, Modic extent, quantitative calcification, segmental mobility, amount of nucleus removed, and graded closure quality were not consistently recorded. Limited non-overlap also remained in the propensity-score tails, and no overlap-restricted analysis was performed.

Fifth, complication-specific variables were not collected systematically. Dural tear, neurological deterioration, dysesthesia, infection, hematoma, and suture-related compression may have been incompletely documented. Reliable incidence estimates and comparative safety analyses could not therefore be performed.

Sixth, the original independent Pfirrmann ratings were not retained; only consensus grades were available. Inter-rater agreement could not be calculated, and misclassification between adjacent grades may have attenuated observed associations. In addition, the reasons for absent documented repair in 25 patients could not be reliably separated into repair not attempted, technical unsuitability, or unsuccessful attempted repair. The study therefore cannot determine whether Pfirrmann grade influences candidacy, repairability, or successful closure. Its conclusions apply only to selected patients in whom repair was successfully completed and should not be generalized to grade V degeneration, marked disc-space collapse, frank instability, severe calcification, or defects that cannot be effectively repaired. Prospective multicenter studies with longer follow-up, systematic imaging, standardized safety reporting, and prospective recording of defect and repair characteristics are required.

## Conclusions

Among selected patients with single-level LDH in whom annular suture repair was successfully completed, Pfirrmann grade IV was not associated with a clinically important disadvantage in 12-month pain or functional outcomes compared with grades I–III. No increased signal of MRI-confirmed symptomatic recurrence was observed, but recurrence analyses were limited by eight events and symptom-triggered MRI. These findings do not determine whether Pfirrmann grade influences surgical candidacy, repairability, or successful annular closure.

## Data Availability

The de-identified patient-level data supporting the findings of this study are not publicly available because they contain potentially sensitive clinical information. The data may be made available by the corresponding author upon reasonable request, subject to approval by the institutional ethics committee and compliance with applicable institutional data-protection requirements.
