# Supplementary material for: Association of preoperative Pfirrmann grade with 12-month outcomes after interlaminar full-endoscopic lumbar discectomy with successful annular suture repair: a single-center retrospective cohort study
Source: Front Surg. 2026 Oct 8;13:1924468. doi: 10.3389/fsurg.2026.1924468 (PMC13686989; doi:10.3389/fsurg.2026.1924468)
Supplement: Supplementary file 1 [file Datasheet1.docx]

**Supplementary Table S1. Covariate balance before and after ATT-IPTW weighting using the expanded propensity-score model**

| Covariate | Before weighting SMD | After ATT-IPTW SMD |
| --- | --- | --- |
| Age, years | 0.623 | 0.027 |
| Female sex | 0.370 | 0.009 |
| BMI, kg/m² | 0.084 | 0.037 |
| Symptom duration, months | 0.043 | 0.021 |
| Current smoker | 0.003 | 0.013 |
| Diabetes mellitus | 0.138 | 0.070 |
| Hypertension | 0.208 | 0.058 |
| Index level L4/5 | 0.059 | 0.017 |
| Right-sided herniation | 0.035 | 0.034 |
| DHI | 0.073 | 0.058 |
| Herniation morphology: protrusion vs extrusion/sequestration | 0.152 | 0.009 |
| Modic type I | 0.154 | 0.062 |
| Modic type II/III | 0.358 | 0.040 |
| Preoperative VAS-back | 0.184 | 0.057 |
| Preoperative VAS-leg | 0.073 | 0.022 |
| Preoperative ODI | 0.050 | 0.035 |

Note. Values are absolute standardized mean differences. The propensity-score model included age, sex, BMI, symptom duration, smoking status, diabetes mellitus, hypertension, index surgical level, herniation laterality, DHI, herniation morphology (protrusion vs extrusion/sequestration), Modic type (absent, type I, and type II/III), preoperative VAS-back, preoperative VAS-leg, and preoperative ODI. Raw ATT weights were 1 for Pfirrmann grade IV and e/(1 − e) for Pfirrmann grades I–III. All post-weighting absolute SMDs were < 0.10; the maximum residual SMD was 0.070 for diabetes mellitus.

Abbreviations: ATT, average treatment effect on the treated; IPTW, inverse probability of treatment weighting; SMD, standardized mean difference; DHI, disc height index; VAS, visual analogue scale; ODI, Oswestry Disability Index.

**Supplementary Figure S1. Propensity-score overlap, ATT weight distribution, and covariate balance before and after ATT-IPTW.**
(A) Distribution of estimated propensity scores in patients with Pfirrmann grades I–III and Pfirrmann grade IV. The shaded region indicates the empirical common-support interval (0.049–0.729). (B) Distribution of raw ATT weights among patients with Pfirrmann grades I–III; patients with Pfirrmann grade IV received a weight of 1 by definition, as indicated by the dashed vertical line. (C) Love plot showing absolute standardized mean differences before and after ATT-IPTW weighting for all covariates included in the expanded propensity-score model. The dashed vertical line at an absolute SMD of 0.10 represents the prespecified threshold for adequate covariate balance. ATT-IPTW, inverse probability of treatment weighting for the average treatment effect in the treated; SMD, standardized mean difference.

**Supplementary Table S2. Comparison of available baseline characteristics between patients included in the complete-case analytic cohort and patients who did not complete 12-month follow-up**

| Characteristic | Included (n = 226) | Did not complete 12-month follow-up (n = 54) | P value |
| --- | --- | --- | --- |
| Age, years | 51.0 (38.0–56.0) | 49.0 (35.5–56.0) | 0.578 |
| Female sex, n (%) | 99 (43.8) | 25 (46.3) | 0.762 |
| Index level L4/5, n (%) | 134 (59.3) | 26 (48.1) | 0.168 |
| Pfirrmann grade I/II/III/IV, n | 2/8/141/75 | 0/3/35/16 | — |
| Pfirrmann grade IV, n (%) | 75 (33.2) | 16 (29.6) | 0.616 |
| Preoperative VAS-back | 5.04 ± 0.93 | 5.04 ± 0.64 | 0.988 |
| Preoperative VAS-leg | 7.08 ± 1.13 | 7.06 ± 1.04 | 0.880 |
| Preoperative ODI | 51.59 ± 8.72 | 50.22± 7.46 | 0.244 |

Note. Continuous variables are presented as mean ± SD or median (interquartile range), as appropriate; categorical variables are presented as n (%). Preoperative VAS-back, VAS-leg, and ODI were available for all 226 included patients and all 54 patients who did not complete 12-month follow-up. P values for these three outcomes were calculated using Welch’s t test. The absolute standardized mean difference for Pfirrmann grade IV was 0.077. ODI, Oswestry Disability Index; SD, standard deviation; SMD, standardized mean difference; VAS, visual analogue scale.
